# Photobiological Neuromodulation of Resting-State EEG and Steady-State Visual-Evoked Potentials by 40 Hz Violet Light Optical Stimulation in Healthy Individuals

**DOI:** 10.3390/jpm11060557

**Published:** 2021-06-15

**Authors:** Yoshihiro Noda, Mayuko Takano, Motoshi Hayano, Xuemei Li, Masataka Wada, Shinichiro Nakajima, Masaru Mimura, Shinichiro Kondo, Kazuo Tsubota

**Affiliations:** 1 Department of Neuropsychiatry, Keio University School of Medicine, Tokyo 160-8582, Japan; mayuko.takano@keio.jp (M.T.); mhayano@keio.jp (M.H.); lixmovo@gmail.com (X.L.); masa.wada0622@gmail.com (M.W.); shinichiro.l.nakajima@gmail.com (S.N.); mimura@a7.keio.jp (M.M.); 2 Department of Ophthalmology, Keio University School of Medicine, Tokyo 160-8582, Japan; tsubota@z3.keio.jp; 3 Faculty of Science and Technology, Keio University, Yokohama 223-0061, Japan; 4 Tsubota Laboratory, Inc., Tokyo 160-0016, Japan; kondo@tsubota-lab.com

**Keywords:** photobiological neuromodulation, violet light, steady-state gamma-frequency stimulation, resting-state electroencephalography (EEG), visual-evoked potential, alpha-phase and gamma-amplitude coupling

## Abstract

Photobiological neuromodulation and its clinical application has been investigated in recent years. The response of the gamma-oscillation to human visual stimuli is known to be both burst and resonant in nature, and the coupling between alpha and gamma oscillations may play a functional role in visual processing. To date, there is no study that examined the effects of gamma-frequency violet light (VL) stimulation on human electroencephalography (EEG). In this study, we investigated the neurophysiological changes induced by light stimulation using EEG. The purpose of this study was to evaluate the specific effects of 40 Hz gamma-frequency VL stimulation on EEG activity by comparing the effects of white light (WL) with the same condition. Twenty healthy participants (10 females: 37.5 ± 14.3 years; 10 males: 38.0 ± 13.3 years) participated in this study and the following results were observed. First, when compared with the power spectrum density (PSD) of baseline EEG, 40 Hz-WL induced significant increase of PSD in theta band. Second, compared the PSDs between EEG with 40 Hz-VL and EEG with 40 Hz-WL, 40 Hz-VL induced significantly lower enhancement in delta and theta bands than 40 Hz-WL. Third, when focused on the occipital area, negative peak of VEP with 40 Hz-VL was smaller than that of 40 Hz-WL. Fourth, 40 Hz-VL induced an increase of alpha-gamma coupling during the VEP at the F5 electrode site as well as post-EEG at the C4 electrode site, compared with baseline EEG. Thus, the present study suggested that 40 Hz-VL stimulation may induce unique photobiological neuromodulations on human EEG activity.

## 1. Introduction

### 1.1. Photobiological Neuromodulation

Vision is based on the interaction of light with photosensitive receptors in the retina. Once light is absorbed by these receptors, it causes photochemical reactions, and the light energy is converted into electrical signals. Photobiological neuromodulation is a concept that describes the comprehensive biophysical modifying effects of light energy. Furthermore, clinical applications using the light sources in non-ionizing forms, such as lasers, LEDs, and broadband light, have been investigated in recent years. Photobiological neuromodulation is a non-thermal process that induces photophysical and photochemical events at various biological scales. Indeed, since light is a powerful stimulus for regulating circadian rhythms, hormones, and behavioral systems, a conventional light therapy is effective in treating seasonal affective disorders, and sleep disorders including circadian rhythm sleep disorder. Moreover, light induces a complex chain of physiological reactions in tissues through its photophysical and photochemical effects, which in turn helps to restore normal cellular functions by promoting blood circulation and reducing inflammation [1] by way of distinct photoreceptors in the retina such as melanopsin or neuropsin expressed in retinal ganglion cells other than the traditional rods and cones [2]. As such, photobiological neuromodulation therapy for brain has potential to enhance the metabolic capacity of neurons and stimulate anti-inflammatory, anti-apoptotic, and antioxidant responses, neurogenesis, and synapse formation, and thus it is expected to have therapeutic applications for central nervous system diseases including depression and dementia [3].

### 1.2. Melanopsin/Neuropsin Expressed in Retinal Ganglion Cells and Violet Light (VL)

**Figure 1:**
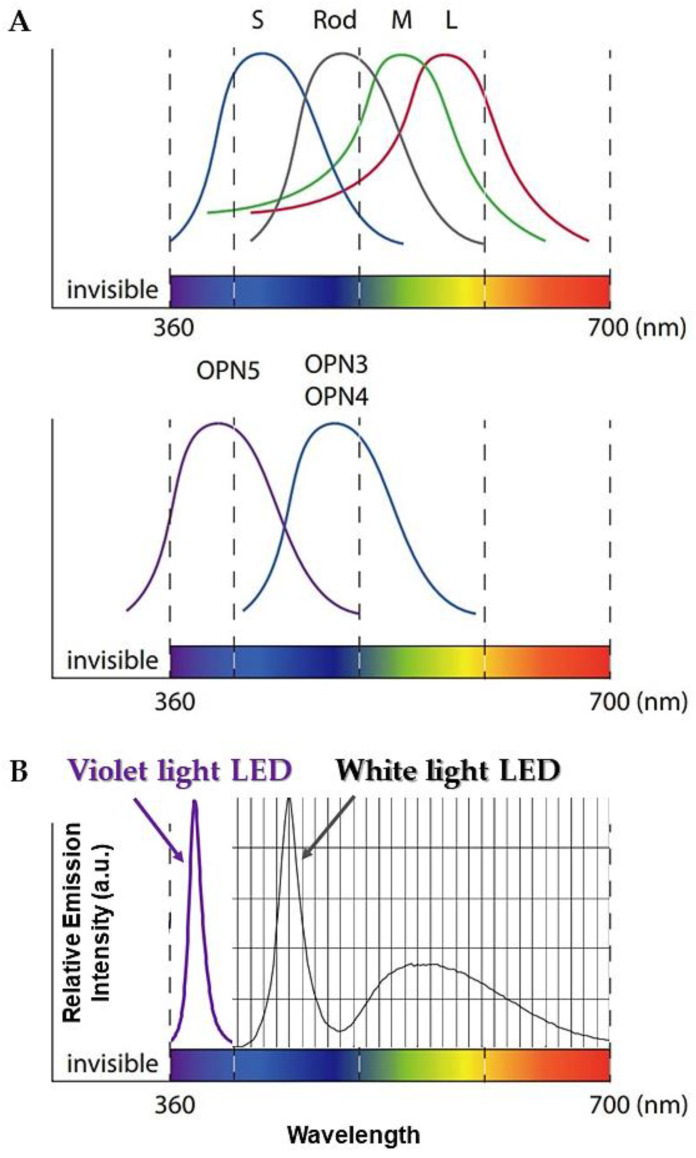
A diagram showing the relationships between the emission spectrum of the LEDs (VL and WL) and the absorption spectrum of the opsins (OPN5, OPN3, and OPN4). (**A**) shows the biological absorption spectrum of opsins; (**B**) represents the emission wavelength of the VL-LED and WL-LED. As shown in Figure 1, the emission wavelength of the VL-LED and the absorption spectrum of OPN5 correspond to each other. VL: violet light; WL: white light; OPN: opsin; S: short-wavelength cone; M: middle-wavelength cone; L: long-wavelength cone.

The signals from retinal ganglion cells project both into the retina and the brain [4] and contain membrane receptors for the neurotransmitters such as glutamate, glycine, and GABA [5]. Of note, photosensitive ganglion cells depolarize on sensing light, thus increasing the rate of neural firings, which is the opposite of other photoreceptor cells that hyperpolarize in response to light [6]. For example, melanopsin acts as an excitable photopigment as well as photoisomerase, which isomerizes all-trans-retinal into 11-cis-retinal by itself when stimulated with light [6]. In contrast, neuropsin is the other photoreceptor protein in neural tissues encoded by the OPN5 gene [7,8] and it is sensitive to VL. Human neuropsin, which is expressed in the eyes, brain, and spinal cord, can activate the heterotrimeric G protein Gi-mediated pathway [9,10]. Furthermore, there are more than five types of retinal ganglion cells in the brain, and their different processing in the central nervous system may result in the diversity of brain functioning [11]. In addition, since each of these various types of retinal ganglion cells has a different central projection pattern, their diversity may result in complex regulatory mechanisms for circadian and neurophysiological responses [12,13,14]. In mice, OPN5 is also expressed in the hypothalamic preoptic area (POA) as a violet light-sensitive deep brain photoreceptor other than in the retina and skin. Indeed, direct measurements of intracellular cAMP showed that OPN5 POA neurons increase cAMP when stimulated with VL [15]. In addition, VL exposure leads to up-regulate EGR-1, which is a transcriptional regulator, expression in the chick model [16]. For example, the induction of EGR-1 is associated with neuronal activity such as neuronal plasticity and memory formation [17]. More specifically, EGR-1 is used in programming the distribution of methylation sites on brain DNA during brain development, in learning and in long-term neuronal plasticity. Furthermore, VL exposure induces significantly higher up-regulation of EGR1 than blue light and EGR1 mRNA expression varies according to the strength of VL. VL has a wavelength between approximately 360 and 400 nm, referring to the color of any different single wavelength of light on the short-wavelength end of the visible spectrum (see Figure 1). Since human vision is relatively insensitive to these wavelengths, violet colors often appear dark. In recent years, in addition to the lack of ultraviolet (UV) light from light sources such as fluorescent lamps and LEDs, exposure to VL has become relatively low in modern society because many windows, glasses, and contact lenses are now UV protected. Furthermore, especially at this time of the year when the COVID-19 pandemic is forcing people to limit their outings, VL exposure is currently overwhelmingly inadequate.

### 1.3. Clinical Applicability of Non-Visual Stimulation of Light

Again, the most influential aspect of the non-visual stimulation is light-induced phase resetting of endogenous circadian clocks. Because circadian rhythmicity is a feature of nearly every physiological, metabolic, and behavioral system, this phenomenon brings a wide array of biological processes under indirect retinal control. Thus, light acts as a neurophysiological stimulant, increasing subjective and objective measures of alertness and psychomotor reaction time, and reducing lapses of attention [2]. Light has been shown to have antidepressant properties, especially in the treatment of seasonal affective disorder. Furthermore, appropriately timed light exposure can be a non-invasive treatment for circadian rhythm sleep disorders and circadian rhythm disturbances associated with jet lag, shift work, and space flight. In recent years, light therapy has shown promise as a treatment for non-seasonal depression, problems associated with the menstrual cycle, bulimia nervosa, and cognitive problems associated with dementia [18]. More surprisingly, a recent basic study showed that driving fast-spiking parvalbumin-positive interneurons with a gamma-rhythm (40 Hz) reduced the levels of amyloid beta Aβ1-40 and Aβ1-42 [19]. Furthermore, the study showed that non-invasive 40 Hz light-flickering stimulation reduced the accumulation of Aβ1-40 and Aβ1-42 in the visual cortex of the mouse model of Alzheimer’s disease (AD) before the onset of symptoms, and reduced plaque burden in the mouse model of AD even after the onset of symptoms, which suggested that gamma-rhythm stimulation may induce both neuronal and glial responses and alleviate the pathology of AD [19].

### 1.4. Potential Roles of Gamma Oscillations in Visual Stimulation and Visual Pathways

Gamma oscillations consist of interactions between excitatory and inhibitory neurons, resulting in rhythmic inhibition capable of entraining firing within local cortical circuits. Several mechanisms have been reported in which gamma oscillations could act on cortical circuits to modulate their responses to inputs, alter their patterns of activity, and enhance the efficacy of their outputs to downstream targets. Recently, it has been confirmed that optogenetic manipulation of gamma oscillations in the neocortex of animals can alter their behavior. Thus, since gamma oscillations are thought to modulate cortical circuit function, it is necessary to clarify the physiological correlates associated with their specific gamma mechanism in the future. Therefore, it is important to elucidate the role of gamma oscillations on cortical circuit functions in normal and pathological conditions through EEG measurements [20]. Furthermore, gamma-rhythm has been associated with high-level cognitive functions such as attention and feature binding and has been reported to be abnormal in brain disorders such as autism and schizophrenia [21]. On the other hand, long-range gamma-band EEG oscillations are known to mediate information transfer between distant brain regions. Gamma-band-based coupling may be used not only for intercortical communication but also in non-cortical areas related to visual pathways. Evoked potentials associated with retinal inputs and the visual cortex show temporally synchronized gamma coherence, which suggests gamma oscillatory coupling between the retina and the visual cortex [22]. Moreover, gamma-band response to visual stimulation exhibits both burst and resonant properties. For example, the photic driving stimulation with alpha-frequency band often produces not only strong alpha entrainment but also significant amplitude modulation of gamma-frequency activity. Conversely, alpha amplitude modulation may occur when gamma-rhythm photic stimulation induces transient alpha rhythm activity and subsequent its suppression. Thus, alpha- and gamma-band activity may be neuromodulated in response to visual stimuli, and it is known that there is a mutual interaction between alpha- and gamma-rhythm generating systems [23].

### 1.5. EEG as a Means of Detecting Representations in the Brain

Since Hans Berger first measured the bioelectrical activity of the human brain non-invasively in 1929, EEG has been developed and established as a tool to monitor brain dynamics and brain functions. In particular, EEG is a modality that can directly evaluate brain activity and has high temporal resolution, making it suitable for neurophysiological assessment that reflects representations in the brain. In recent years, high-density EEG system with multiple channels has become common and both spontaneous and evoked activities have been of interest. In EEG recordings, undesired signals or artifacts other than EEG can be divided into two main categories: physiological and non-physiological. Physiological artifacts are electrical signals generated by the heart, muscles, especially eye and facial muscles, and retina. These artifacts, which can be several orders of magnitude larger than the EEG signal of interest, usually have a characteristic topography and can be reduced by sophisticated spatial artifact removal techniques such as ICA [24].

### 1.6. Visual-Evoked Potentials (VEPs) and Cross-Frequency Coupling (CFC) of Alpha-Phase and Gamma-Amplitude

Visual-evoked potentials (VEPs) are the brain’s response to repetitive visual stimuli, and their high signal-to-noise ratio and ability to entrain oscillatory brain activity are useful for investigating the neural processes underlying rhythmic activity in the brain, and for elucidating the causal role of brain rhythms in cognition and emotion. A previous study examined oscillatory EEG dynamics in the time-frequency domain of VEP induced by visual stimulation with gamma-frequency (40–60 Hz). Gamma-frequency visual stimulation induced VEPs at the parieto-occipital region, and transient responses were accompanied by increases in frontal and occipital delta and theta power between 0 and 300 ms after stimulus onset, returning to baseline after about 500 ms. Furthermore, occipital beta/gamma-band power enhanced during the visual stimulation period, which may have been due to increased power at the sub-harmonic frequency of the stimulus [25]. Thus, cross-frequency coupling (CFC) has been suggested to be a highly flexible mechanism for information gating and processing in the cerebral cortex, giving rise to a wide range of higher-order cognitive functions in humans. Specifically, CFC may be an elegant mechanism for integrating information across multiple spatiotemporal scales in coupled neuronal networks. Indeed, a previous study has demonstrated that selective entrainment of alpha/gamma oscillations induced specific neuromodulation on CFC, which indicated that entrainment of low-frequency components (e.g., alpha band activity) increased phase-amplitude coupling, especially gamma-band power, which became preferentially locked to alpha oscillations, whereas entrainment of the gamma-band resulted in a decrease in alpha power. These reciprocal influencing results indicate that the coupling between alpha and gamma oscillations may play a functionally important role in visual processing [26].

### 1.7. Objectives and Hypotheses of the Present Study

To date, no study has evaluated the effects of combined 40 Hz gamma-frequency violet light (VL) stimulation on human EEG. Thus, in the present study, we aimed to investigate the neurophysiological effects of 40 Hz gamma-frequency VL stimulation on human resting EEG activity as well as steady-state visual-evoked potentials (VEPs) by comparing the effects of wavelengths of white light (WL). Specifically, we hypothesized that 40 Hz gamma-frequency VL stimulation would cause photobiological neuromodulation specific to the alpha and gamma activity generating systems, as represented by the coupling between alpha-phase and gamma-amplitude.

## 2. Materials and Methods

### 2.1. Participants

Twenty healthy individuals participated in this study. Participants were eligible to participate in this study if they met the following criteria: (i) over 20 years old; (ii) no current or history of cardiovascular, hepatic, endocrine, cerebral/neurological, or psychiatric diseases that may affect the study results; (iii) no prescription medications; (iv) no history of alcohol or other drug abuse/dependence; and (v) no history of photosensitive seizures or family history of photosensitive seizures. Participants were screened by the research group to ensure that they met the above inclusion criteria. The present study was conducted according to the Declaration of Helsinki and was reviewed and approved by the Ethics Committee of Keio University School of Medicine (Shinjuku-ku, Tokyo, Japan; ID: 20190096). In this experiment, the investigators also interviewed the participants to confirm the safety and adverse events during and after the light stimulation.

### 2.2. Experimental Design

**Figure 2:**
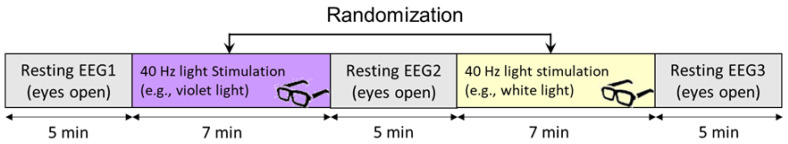
A schematic diagram of the experiment in this study.

This experiment was conducted in a crossover design. Specifically, we measured resting-state EEG for 5 min as baseline, 7 min of EEG during light stimulation (violet light (VL) or white light (WL)), 5 min of resting-state EEG after light stimulation, then 7 min of EEG during light stimulation by switching the light stimulation condition (VL or WL), and 5 min of resting EEG after the light stimulation, for a total of approximately 30 min. The order of WL and VL stimulation to the participants was completely randomized based on a random number table to avoid bias. A schematic diagram of the experiment is shown in Figure 2.

### 2.3. Violet Light (VL) and White Light (WL) Stimulation

**Figure 3:**
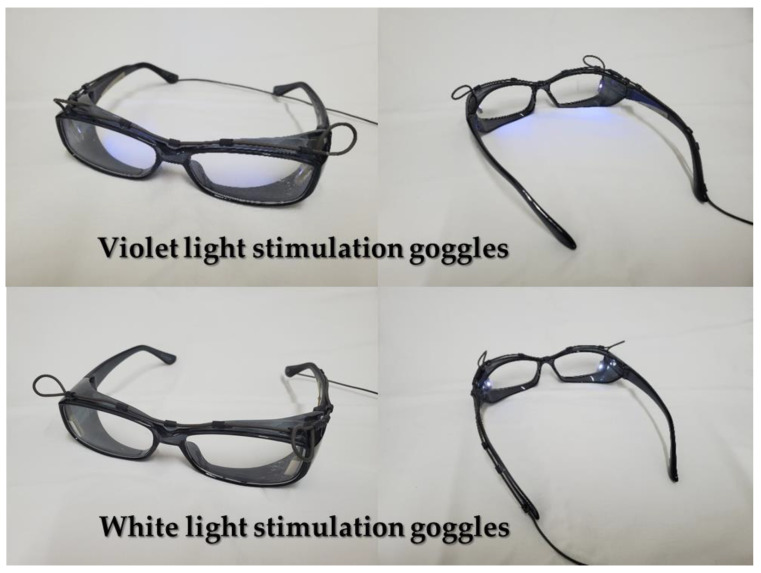
Goggles for violet light (VL) stimulation and goggles for white light (WL) stimulation used in this experiment.

EEG was measured before, during, and after exposure to different light conditions, using steady-state 40 Hz-VL (375 nm) and steady-state 40 Hz-WL with 2-s interval flicker stimulation in healthy adults. For the VL used for optical stimulation, we use goggles with a commercially available LED light source module (VL-LED: NSSU123; Nichia Corporation, Anan, Tokushima, Japan; peak wavelength 375 nm) with a circuit for pulse width modulation (PWM; modulation frequency kHz). The UV radiation from this LED is 310 µW/cm^2^. For the WL, we used separate goggles equipped with a commercial LED light source module with a circuit for PWM (WL-LED: NSSW157T; Nichia Corporation, Anan, Tokushima, Japan). Regarding the intensity of VL and WL conditions, we did not make them identical in terms of physical intensity but adjusted them to be as equivalent as possible in brightness at the perceptual level seen by the human eyes. Specifically, in the preparation stage of this research protocol, three scientists (Y.N., X.L., and M.H.) in our research group adjusted the brightness of the VL and WL multiple times so that the perception of the brightness of both conditions would be almost identical. Furthermore, the reason we matched the brightness of VL and WL at the perceptual level in this way was that if we unified the brightness at the physical intensity, the energy of VL becomes relatively higher than that of WL due to the wavelength and frequency of each light. In addition, if we match the brightness with the physical intensity, the brightness of the light sources in VL and WL will be obviously different, which may eliminate the blindness of the participants regarding the type of light and may introduce psychological placebo effect into the light stimulation experiment. See Figure 3 for photos of these goggles.

### 2.4. EEG Recording

EEG was recorded through a 62-channel TruScan LT with an EEG cap (DEYMED Diagnostic s.r.o., Hronov, Czech Republic). During the EEG recording, electrodes impedance was kept below 5 kΩ. All electrodes were referenced to an average of both earlobes’ electrodes (A1 and A2). EEG signals were recorded at a sampling rate of 3000 Hz. Since the main purpose of this study was to precisely investigate the neurophysiological effects induced by violet light stimulation through the retina on EEG, we used a high-resolution EEG system.

### 2.5. EEG Signal Preprocessing

EEG data were processed offline using the MATLAB software (R2020a, The MathWorks, Natick, MA, USA) and EEGLAB toolbox (Swartz Center for Computational Neuroscience, University of California at San Diego, CA, USA). First, continuous EEG data were segmented every 2-s, and then the EEG data during visual light stimulation were epoched from −1000 ms to 2000 ms based on the trigger signal of the first light stimulus (see Supplementary Materials). Furthermore, the baseline correction was performed using the pre-stimulus interval between −500 ms and −100 ms. Then, these EEG data were referenced to the average reference. Next, the Butterworth 0.5–70 Hz bandpass filter and notch filter (48–52 Hz: this is because in eastern Japan, the frequency of power line noise is 50 Hz) were applied. Afterwards, the preprocessed data were downsampled to 1000 Hz. Furthermore, the preprocessed EEG data were visually inspected to exclude segments that were highly contaminated with artifacts. Here, there was no need to remove the bad channels because the quality of the EEG data was maintained in this study. Subsequently, independent component analysis (ICA) was applied to minimize and remove the eye-related and muscle activity related components. In each participant, the number of ICA components that were removed from the original 62 components was no greater than 20%.

### 2.6. Power Spectrum Density (PSD) Analysis

The power spectrum of a time series describes the distribution of power to the frequency components that comprise the signal. The PSD refers to the spectral energy distribution found per unit time. Fourier transform was applied to the preprocessed EEG data to compute the PSD. In the present analysis, the average PSDs of all electrodes (62 channels), frontal region (Fp1, Fp2, Fpz, AF7, AF3, AFz, AF4, and AF8), and occipital region (O1, O2, Oz, PO7, PO3, POz, PO4, and PO8) were calculated for each delta (1–3 Hz), theta (4–7 Hz), alpha (8–13 Hz), beta (14–30 Hz), and gamma (30–70 Hz) frequency bands.

### 2.7. Visual-Evoked Potential (VEP) Analysis

VEP is an evoked potential elicited by presenting light flash which can be clinically used to examine some visual pathway damage including retina, optic nerve, optic chiasm, optic radiations, and occipital cortex. As with other evoked potential tests, VEP is conducted in the event-related potential (ERP) paradigm. The 40 Hz photo-stimulation with a time duration of 1 s was flashed at 2-s intervals, and a total of 200 flashes were presented in one condition of photo-stimulation experiment, and then the steady-state VEP was analyzed by summing and averaging those 200 epochs for the first 500 ms during light stimulation.

### 2.8. Phase-Amplitude Coupling (PAC) Analysis during the VEP

First, EEG signal was filtered into separate delta, theta, alpha, and beta band waveforms for phase and theta, alpha, beta, and gamma waveforms for amplitude with a zero-phase shift filter for all electrodes for 500 ms time windows. Subsequently, the Hilbert transform was applied to the processed EEG data and then time series data of phase and amplitude were calculated. The modulation index (MI) of phase-amplitude coupling was computed as possible relationships between the delta, theta, alpha, and beta band phases and the theta, alpha, beta, and gamma amplitudes based on the previously published method [27]. The specific-amplitudes corresponding to the specific-phase were sorted into 18 bins and then they were averaged. To quantify the coupling, the relative amplitude distribution for each participant was calculated, dividing the amplitude of each phase by the sum of all amplitudes across bins. In other words, the MI was obtained by measuring the divergence of the amplitude group from a uniform distribution.

### 2.9. Statistical Analysis

Statistical analysis was conducted by the MATLAB software (R2020a, The MathWorks, Natick, MA, USA). For demographic data, an independent *t*-test was applied to compare age difference between female and male participants. For the EEG analysis data, permutation tests with 10,000 permutation samples were applied to compare the difference in PSDs among the conditions. In addition, exploratory correlation analyses were performed between the VEP peak and PSD of each frequency band as well as the MI values for significant changes of phase-amplitude couplings and PSD of each frequency band. In the present study, we set the significance level at 0.05 and did not correct for multiple comparisons, since these were preliminary hypothesis-testing analyses as described in the introduction.

## 3. Results

### 3.1. Demographic Data of the Participants

In the present study, 20 healthy individuals (10 females and 10 males) were participated. The mean age (±S.D.) of the participants was 37.8 ± 13.4 years (females: 37.5 ± 14.3 years; males: 38.0 ± 13.3 years). There was no significant difference in age between females and males (*t*_18_ = −0.081, *p* = 0.936). The breakdown of participants who were preceded by 40 Hz-WL stimulation and followed by 40 Hz-VL stimulation was as follows: the number of participants was 10 (4 females and 6 males) with a mean age (±S.D.) of 37.9 ± 11.7 years. In contrast, the number of participants who were preceded by 40 Hz-VL stimulation and followed by 40 Hz-WL stimulation was 10 (6 females and 4 males) with a mean age (±S.D.) of 37.6 ± 15.6 years. There was no significant difference in sex (*χ*^2^ = 0.800, *p* = 0.371) and age (*t*_18_ = −0.049, *p* = 0.962) between the above-mentioned attributes for the different order of light stimulation. Of note, no obvious adverse events were reported during or after the light stimulation experiments in this study, such as eye or skin damage, visual abnormalities, headache, discomfort, or nausea.

### 3.2. PSD Analysis

#### 3.2.1. Averaged PSDs over All Electrodes for Baseline EEG, during, and after the Light Stimulations

**Figure 4:**
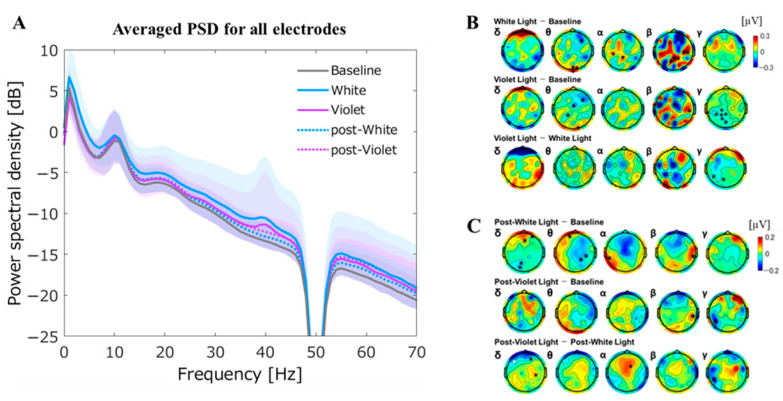
Averaged PSDs for all electrodes during and after the light stimulation and their topographical plots. (**A**) shows the averaged power spectrum density (PSD) over all electrodes for each condition; (**B**) depicts topographical plots of PSDs during the light stimulation; (**C**) demonstrated topographical plots of PSDs after the light stimulation. Of note, black asterisks indicate significant findings at the 0.05 level of significance, while white asterisks indicate significant findings at the 0.01 level of significance. The graph is shown with ±S.D. shadows to the mean PSD for each condition.

Compared with the averaged PSD over the all electrodes of baseline EEG, the averaged PSD for all electrodes with 40 Hz-WL stimulation showed a significant increase in theta band. Furthermore, when compared the averaged PSDs for all electrodes between VEP with 40 Hz-VL and VEP with 40 Hz-WL, there were significant differences in delta and theta bands, indicating that the averaged PSDs for 40 Hz-WL in these bands showed significantly higher powers compared with the averaged PSDs for 40 Hz-VL. In addition, the averaged PSD over the all electrodes for post-EEG with 40 Hz-VL demonstrated a marginally significant increase in gamma-band compared with the averaged PSD of baseline EEG (Figure 4A; Table 1).

#### 3.2.2. Topographical Plots of PSDs during the Light Stimulation

Topographical plots of PSD during the light stimulation are depicted in Figure 4B.

##### PSD of Baseline EEG vs. PSD of VEP with 40 Hz-WL Stimulation

When the topological plots for the PSDs of baseline EEG and VEP paradigm with 40 Hz-WL stimulation are viewed at the electrode level, the PSDs were neuromodulated from theta to beta bands. Specifically, in theta band, 40 Hz-WL stimulation induced reduced PSD over the right prefrontal area and increased PSD over the occipital area; in alpha band, increased PSD at the left central and midline parietal areas; and in beta band, increased PSD at the right temporal and midline parietal areas, compared with baseline EEG (Figure 4B, upper row).

##### PSD of Baseline EEG vs. PSD of VEP with 40 Hz-VL Stimulation

Likewise, in the VEP paradigm with 40 Hz-VL stimulation, PSDs at the electrode level were neuromodulated over the theta, beta, and gamma bands. Specifically, 40 Hz-VL stimulation induced reduced PSD in theta band over the right frontal and midline central areas; reduced PSD at the midline central area and increased PSD at the right temporal area in beta band; and reduced PSD in gamma-band over the midline and left parietal areas (Figure 4B, middle row).

##### PSD of VEP with 40 Hz-WL vs. PSD of VEP with 40 Hz-VL

At the electrode level, the analyses between the PSDs of VEP with 40 Hz-WL and 40 Hz-VL revealed that there was a difference in the PSD in gamma-band at the left parieto-occipital area, indicating that 40 Hz-VL showed a lower gamma modulation on PSD compared with 40 Hz-WL (Figure 4B, lower row).

#### 3.2.3. Topographical Plots of PSD after the Light Stimulation

Topographical plots of PSD after the light stimulation are shown in Figure 4C.

##### PSD of Baseline EEG vs. PSD of Post-EEG with 40 Hz-WL

Compared with PSD of baseline EEG, PSD for post-EEG with 40 Hz-WL stimulation showed partial EEG neuromodulations from the delta to beta bands at the electrode level. Specifically, PSD for post-EEG with 40 Hz-WL increased at the mid-prefrontal while it was reduced at the mid-parieto-occipital areas in delta band; PSD was reduced at the right parietal area in theta band; PSD increased at the left temporal area in alpha band; and PSD increased at the right temporal area in beta band (Figure 4C, upper row).

##### PSD of Baseline EEG vs. PSD of Post-EEG with 40 Hz-VL

Compared with baseline EEG, 40 Hz-VL stimulation induced enhanced PSD in beta band at the right parietal area and reduced PSD in gamma-band at the left parietal area (Figure 4C, middle row).

##### PSD of Post-EEG with 40 Hz-WL vs. PSD of Post-EEG with 40 Hz-VL

When compared the PSDs between VEP with 40 Hz-WL and VEP with 40 Hz-VL at the electrode level, 40 Hz-VL stimulation induced reduced PSD for post-EEG in delta band over the left and right prefrontal areas and increased PSD at the right parietal area; increased PSD in alpha band over the midline central area; and reduced PSD in gamma-band at the left parietal area, compared with 40 Hz-WL stimulation (Figure 4C, lower row).

#### 3.2.4. Averaged PSD for the Prefrontal and Occipital Cortex during the Light Stimulation

**Figure 5:**
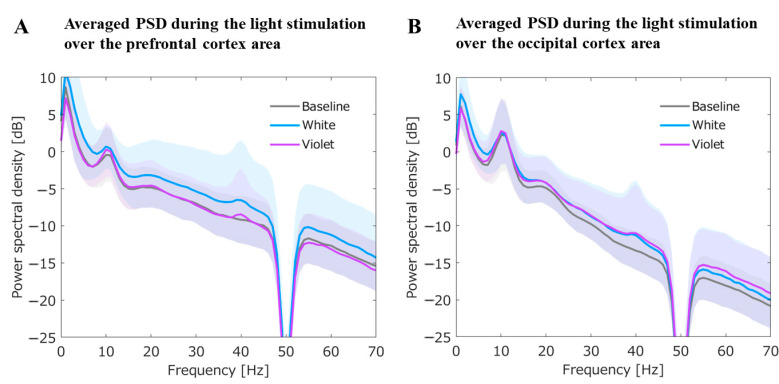
Averaged PSDs for the prefrontal and occipital cortex during the light stimulation. (**A**) shows averaged PSDs for the prefrontal region of interest (ROI) during the light stimulation. Compared with the PSD of baseline EEG, the prefrontal PSD for post-EEG with 40 Hz-WL was significantly increased in theta band (*t*_38_ = 2.03, *p* = 0.04); (**B**) shows averaged PSDs for the occipital ROI during the light stimulation. Compared with the PSD of baseline EEG, the occipital PSD for post-EEG with 40 Hz-WL was significantly increased in delta band (*t*_38_ = 1.98, *p* = 0.04). The graphs are presented with ±S.D. shadows to the mean PSD for each condition.

Averaged PSDs for the prefrontal and occipital cortex during the light stimulation are shown in Figure 5. In addition, statistical results are summarized in Table 2.

##### Averaged PSDs for the Prefrontal ROI during the Light Stimulation

For the PSDs focused on the prefrontal area, the PSD in theta band during the 40 Hz-WL stimulation showed an increase compared with PSDs of baseline EEG and post-EEG with 40 Hz-VL. Additionally, for both the PSDs with 40 Hz-VL and 40 Hz-WL, 40 Hz frequency peaks on PSDs were non-significantly enhanced probably due to the entrainment of this specific gamma-frequency (Figure 5A).

##### Averaged PSDs for the Occipital ROI during the Light Stimulation

The PSD in delta band during the 40 Hz-WL stimulation showed an increase compared with PSDs of baseline EEG and post-EEG with 40 Hz-VL. Likewise, for both the PSDs with 40 Hz-VL and 40 Hz-WL, 40 Hz frequency peaks on PSDs were non-significantly enhanced probably due to the entrainment of this specific gamma-frequency (Figure 5B).

#### 3.2.5. Averaged PSDs for the Prefrontal and Occipital ROIs for Post-EEG with the Light Stimulation

**Figure 6:**
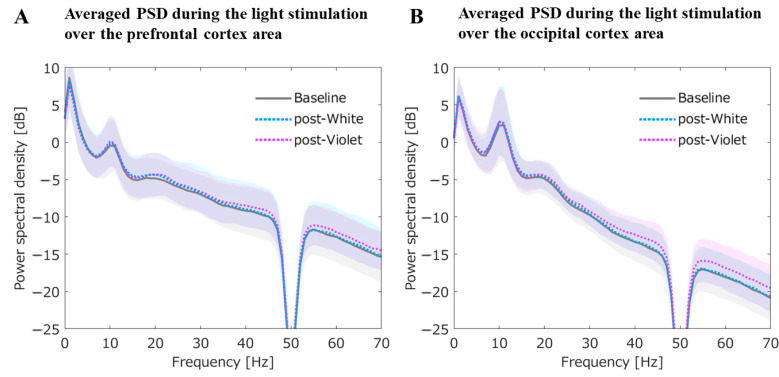
Averaged PSDs for the prefrontal and occipital cortex after the light stimulation. (**A**) shows averaged PSDs for the prefrontal ROI after the light stimulation. When comparing the PSD in the baseline EEG, EEG after 40 Hz-WL, and EEG after 40 Hz-VL conditions, there was no significant difference in any of the frequency bands; (**B**) shows averaged PSDs for the occipital ROI after the light stimulation. There was no significant difference in any of the frequency bands among the three conditions (i.e., baseline EEG, post-EEG with 40 Hz-WL, and post-EEG with 40 Hz-VL). The graphs are presented with ±S.D. shadows to the mean PSD for each condition.

Averaged PSDs for the prefrontal and occipital cortex for post-EEG with the light stimulation are shown in Figure 6. Furthermore, statistical results are summarized in Table 3.

##### Averaged PSDs for the Prefrontal ROI after the Light Stimulation

When compared the PSDs of baseline EEG, post-EEG with 40 Hz-WL, and post-EEG with 40 Hz-VL conditions, there was no significant difference in any of the frequency bands (Figure 6A).

##### Averaged PSDs for the Occipital ROI after the Light Stimulation

In contrast, the analysis focused on the occipital area showed no significant differences in any of the frequency bands among the three conditions (i.e., baseline EEG, post-EEG with 40 Hz-WL, and post-EEG with 40 Hz-VL) (Figure 6B).

#### 3.2.6. VEP at the Occipital Area

**Figure 7:**
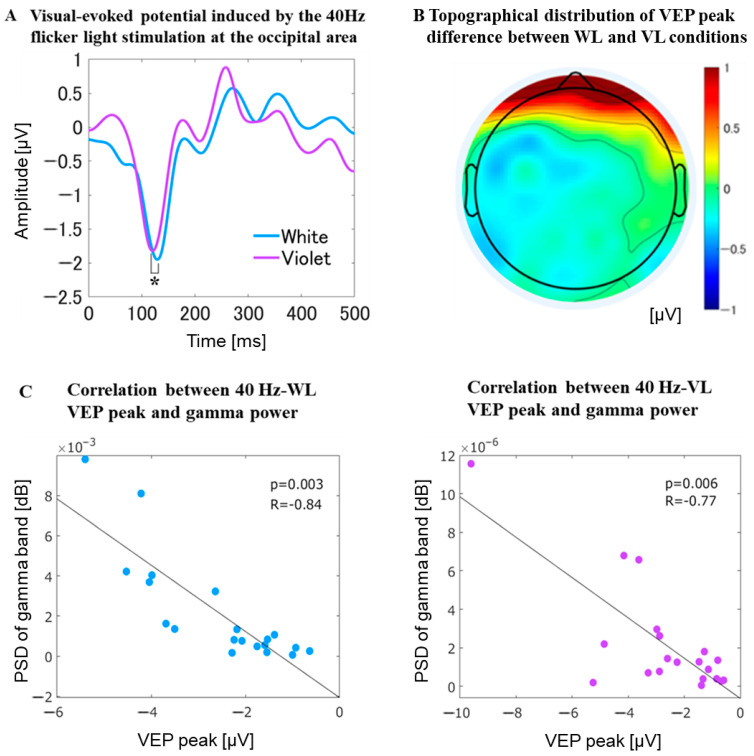
Visual-evoked potential at the occipital area. (**A**) represents the VEP at the occipital area (Oz electrode site). The negative peak of VEP appeared around 100 ms with 40 Hz-WL stimulation, whereas the corresponding negative peak of VEP with 40 Hz-VL was significantly smaller (*t*_38_ = 2.04, *p* = 0.032) than that of 40 Hz-WL. However, there was no significant difference in the negative peak latencies between the VEPs with 40 Hz-VL and the VEP with 40 Hz-WL; (**B**) depicts topographical distribution of VEP peak difference between 40 Hz-WL and 40 Hz-VL stimulation conditions; (**C**) shows significant correlations between VEP peak and PSD of gamma-band during 40 Hz-WL stimulation as well as between VEP peak and PSD of gamma-band during 40 Hz-VL stimulation.

For the VEP at the occipital area (Oz electrode site), the negative peak of VEP appeared around 100 ms with 40 Hz-WL stimulation, whereas the corresponding negative peak of VEP with 40 Hz-VL was significantly smaller (*t*_38_ = 2.04, *p* = 0.032) than that of 40 Hz-WL (Figure 7). However, there was no significant difference in the negative peak latencies between the VEP with 40 Hz-VL and the VEP with 40 Hz-WL. Interestingly, there were significant correlations between VEP peak and PSD of gamma-band during 40 Hz-WL stimulation as well as between VEP peak and PSD of gamma-band during 40 Hz-VL stimulation over the occipital area.

#### 3.2.7. Phase-Amplitude Coupling Analysis during VEP

##### Differences in Phase-Amplitude Coupling between EEG with 40 Hz-WL Stimulation and Baseline EEG as Well as between Post-EEG for 40 Hz-WL Stimulation and Baseline EEG

There were no significant impacts on phase-amplitude coupling by 40 Hz-WL stimulation for all electrodes and for both conditions (VEP during 40 Hz-WL and post-EEG with 40 Hz-WL stimulation).

##### Differences in Phase-Amplitude Coupling between EEG with 40 Hz-VL Stimulation and Baseline EEG as Well as between Post-EEG for 40 Hz-VL Stimulation and Baseline EEG

**Figure 8:**
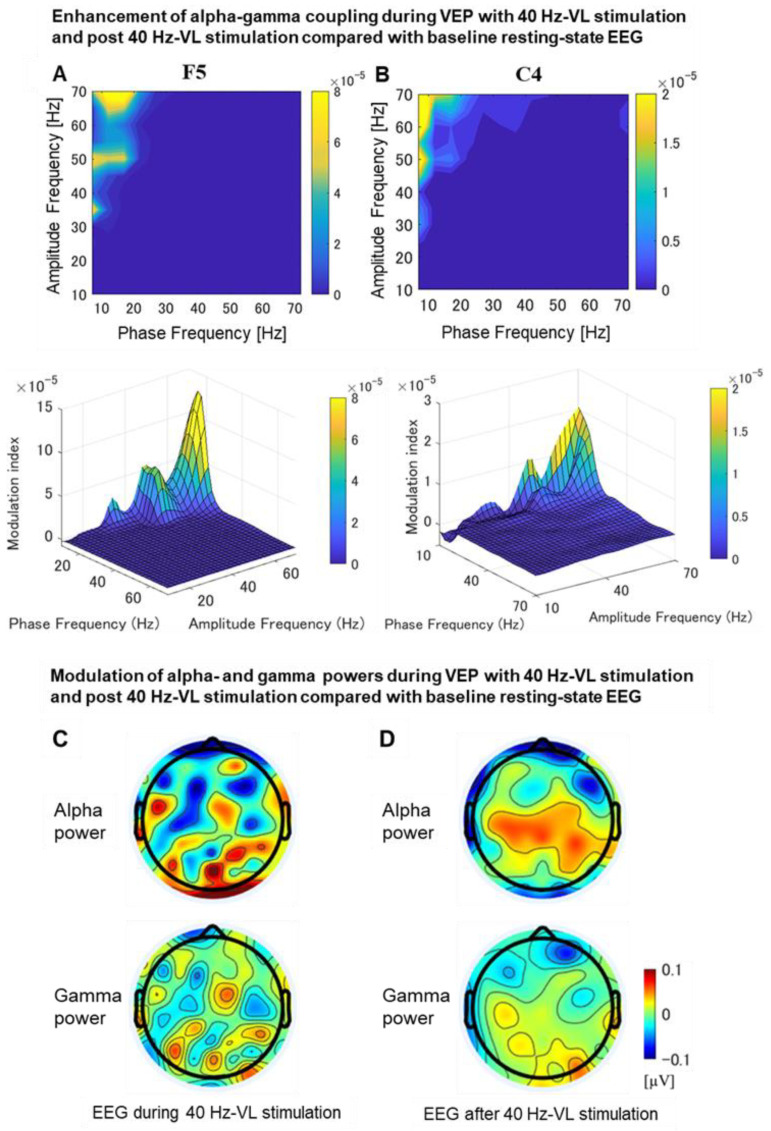
Enhanced alpha-phase and gamma-amplitude coupling and modulation of alpha and gamma power during and after 40 Hz-VL stimulation. (**A**, **B**) show the comodulogram, where the vertical axis represents the amplitude frequency, and the horizontal axis represents the phase frequency. The alpha-phase and gamma-amplitude coupling, which was significant during and after 40 Hz-VL stimulation, corresponds to the area shown in yellow in the color map display on the comodulogram. The upper row are 2-dimensional display graphs, and the lower row are 3-dimensional display graphs (Z-axis is the modulation index value). (**A**) demonstrated a significant increase of alpha-phase and gamma-amplitude coupling at the left prefrontal area (F5 electrode site: *t*_38_ = 2.08, *p* = 0.029) during the 40 Hz-VL stimulation (i.e., VEP with 40 Hz-VL minus baseline EEG); (**B**) indicated a significant increase of alpha-phase and gamma-amplitude coupling at the right central area (C4 electrode site: *t*_38_ = 2.07, *p* = 0.033) after the 40 Hz-VL stimulation (i.e., post-EEG with 40 Hz-VL minus baseline EEG); (**C**) displays EEG topological plots illustrating the changes in alpha- and gamma-band PSDs (0–1000 ms) during 40 Hz-VL stimulation compared to baseline resting-state EEG; (**D**) represents EEG topological plots depicting the changes in alpha- and gamma-band PSDs (0–1000 ms) after 40 Hz-VL stimulation compared to baseline resting-state EEG.

The 40 Hz-VL stimulation induced a significant increase of alpha-phase and gamma-amplitude coupling at the left prefrontal area (F5 electrode site: *t*_38_ = 2.08, *p* = 0.029) among the possible phase and amplitude coupling combinations (Figure 8A). Furthermore, there was a significant increase of alpha-phase and gamma-amplitude coupling at the right central area (C4 electrode site: *t*_38_ = 2.07, *p* = 0.033) after the 40 Hz-VL stimulation as well (Figure 8B).

## 4. Discussion

### 4.1. Summary of Findings

The present study yielded several important findings as follows: (1) compared with averaged PSD of baseline EEG, averaged PSD of 40 Hz-WL in theta band was significantly increased; (2) when compared averaged PSDs between EEG with 40 Hz-VL and EEG with 40 Hz-WL, averaged PSDs for 40 Hz-WL in delta and theta bands showed significantly higher powers compared with averaged PSDs for 40 Hz-VL; (3) when focused on the prefrontal area, the PSD with 40 Hz-VL was significantly lower than PSD with 40 Hz-WL in theta band; (4) when focused on the occipital area, the PSD with 40 Hz-VL was significantly lower than PSD with 40 Hz-WL in delta band; (5) the VEP with 40 Hz-WL showed the negative peak around 100 ms at the Oz electrode site, whereas the VEP with 40 Hz-VL showed a significantly smaller negative peak than that of the VEP with 40 Hz-WL; (6) 40 Hz-VL induced significant increases of alpha-gamma coupling during the VEP at the F5 electrode site as well as for post-EEG with 40 Hz-VL at the C4 electrode site, compared with the coupling of baseline EEG.

### 4.2. Photobiological Neuromodulation of EEG during the 40 Hz-VL Stimulation Compared with 40 Hz-WL Stimulation

During the stimulation, averaged PSD over the all electrodes for EEG with 40 Hz-VL showed significantly lower neuromodulation in delta and theta bands compared with the PSD of EEG with 40 Hz-WL. In addition, averaged PSD over the all electrodes for post-EEG with 40 Hz-VL demonstrated a marginally significant increase in gamma-band compared with the PSD of baseline EEG. Here, since there is a phenomenon in the nervous system called “neural entrainment” in which neural activity is synchronized to the frequency of repetitive external stimuli, it is possible that the whole brain EEG power spectrum at the driving frequency, corresponding to 40 Hz in this study, increased as a steady-state response regardless of the stimulation wavelength of WL or VL [25,28]. Furthermore, when focused on the prefrontal ROI and occipital ROI, the PSD for EEG with 40 Hz-VL over the prefrontal ROI indicated significantly lower neuromodulation in theta band compared with the PSD of EEG with 40 Hz-WL, while the PSD of EEG with 40 Hz-VL over the occipital ROI revealed significantly lower neuromodulation in delta band compared with the PSD of EEG with 40 Hz-WL. Moreover, at the electrode level, when compared with the PSD of baseline EEG, 40 Hz-VL stimulation induced reduced neuromodulations in theta band at the right frontal and midline central areas; reduced neuromodulation in alpha band at the midline central area; reduced neuromodulation at the midline central area and increased neuromodulation at the right temporal area in beta band; and reduced neuromodulation at the midline and left parietal areas in gamma-band. Furthermore, when compared the PSDs between VEP with 40 Hz-WL and VEP with 40 Hz-VL, 40 Hz-VL stimulation induced lower neuromodulations in gamma-band at the left parieto-occipital area, compared with 40 Hz-WL stimulation. In the whole cortex level, both 40 Hz-WL and 40 Hz-VL stimulation tended to induce enhancement of theta oscillation system compared with baseline EEG. Furthermore, 40 Hz-VL stimulation induced a weaker enhancement of PSD in delta and theta bands compared with 40 Hz-WL stimulation. In other words, 40 Hz-VL stimulation may be causing activation of a distinct pathway from the activation of the delta/theta system in the usual visual pathway [29,30]. In addition, 40 Hz-VL stimulation caused local PSD changes compared to the baseline at the electrode level, including broadband neuromodulation from theta to gamma bands. In particular, the attenuative neuromodulation by 40 Hz-VL in the theta, alpha, and gamma bands may reflect unique responses mediated by special pathways, such as OPN5 receptors, that differ from the usual visual pathways. Notably, it is known that there is an interaction between the alpha- and gamma-oscillation generating systems; specifically, activity in the gamma-band induces activity in the alpha band, and furthermore, amplitude modulation occurs by suppressing activity in the alpha system [23]. Taken together, 40 Hz-VL stimulation may be involved in the activation of transient cognitive functions in humans.

### 4.3. Significant Difference in the Negative Peak of the VEPs between 40 Hz-VL and 40 Hz-WL

Both 40 Hz-WL and 40 Hz-VL stimulation evoked a typical VEP pattern in the occipital region. Indeed, it is known that gamma-frequency visual stimulation with WL can induce the typical VEPs at the parieto-occipital region [25]. However, in the present results, the VEP by 40 Hz-VL stimulation was significantly lower than VEP induced by 40 Hz-WL stimulation. This finding suggests that 40 Hz-VL stimulation, unlike 40 Hz-WL, may activate different circuits such as non-visual processing circuits via VL stimulus-OPN5 receptor system. In addition, since there were significant correlations between the VEP peak and PSD of gamma-band in both conditions, during 40 Hz-WL and 40 Hz-VL stimulation, light stimulation at 40 Hz can induce entrainment of the gamma-frequency band in the EEG activity at the occipital region.

### 4.4. Significant Enhancement of Alpha-Gamma Coupling during and after 40 Hz-VL Stimulation

The present study demonstrated that 40 Hz-VL stimulation induced significant increase of alpha-gamma coupling at the left prefrontal area during the stimulation period while it induced the alpha-gamma coupling over the right central area after the stimulation compared to resting-state EEG. Brain oscillations are one of the core mechanisms underlying cognitive functions, including episodic memory, which have also been considered to be important for long-term memory since they induce synchronized firing between cell assemblies that form synaptic plasticity. Indeed, several previous studies have focused on the role of synchronization in episodic memory, as reflected in increases in theta rhythm (∼5 Hz) and gamma-rhythm (>40 Hz) power. However, recent studies have also focused not only on changes in theta and gamma power, but also on the importance of coupling between desynchronization of basic rhythms in the neocortex as represented by alpha rhythm power and synchronization in the gamma-rhythm in the process of cognitive functioning [31,32]. For example, successful encoding and recall of episodic memories requires the ability to (1) represent detailed multisensory information and (2) combine that information into a coherent episode. Previous studies suggest that these two cognitive processes are achieved by desynchronization of alpha rhythms in the neocortex (i.e., decrease in alpha power) and synchronization of gamma rhythms in the hippocampus (i.e., increase in gamma power), respectively. In other words, it has been shown that the co-existence and interaction of these two processes may be important for cognitive processing [33]. As such, the present findings regarding the alpha-gamma coupling during and following the 40 Hz-VL stimulation may represent a potential neurophysiological enhancement of cognitive function.

### 4.5. Limitations of This Study

There are some limitations in this study. First, due to the nature of the study, which was primarily intended for a preliminary hypothesis-based analysis, the sample size of the participants was relatively small, and the significance level was set at 0.05 in this study. Therefore, the results of this study need further validation in the future. Second, since this was a non-interventional experiment with healthy participants, we did not evaluate the effects of 40 Hz-VL light stimulation on cognitive functions and clinical symptoms. Future interventional studies in patient groups compared to controls are needed. Third, since we examined the neurophysiological changes of EEG during and immediately after the light stimulation, the long-term effects after the light stimulation were not assessed. Therefore, future studies need to include long-term follow-up examinations after the light stimulation.

## 5. Conclusions

Collectively, the present study demonstrated that 40 Hz-VL stimulation induced unique photobiological neuromodulations of resting EEG activity as well as the steady-state VEP in healthy participants as represented by alpha-gamma coupling. These neurophysiological modulations suggest that the 40 Hz-VL with stimulation may have some potential proactive effects on cognitive processes in humans. In the future, clinical trials using 40 Hz-VL stimulation in patients with major depression and mild cognitive impairment should be conducted to rigorously confirm whether this 40 Hz-VL optical neuromodulation is actually safe and has beneficial effects on clinical symptoms and cognitive function. Indeed, we are planning to start those clinical trials by the end of 2021.

## Figures and Tables

**Table 1:** Summary of statistical analysis results comparing all electrode averaged PSD between each condition for each frequency band (* *p* < 0.05).

Frequency Band	Baseline vs. 40 Hz-WL		Baseline vs. 40 Hz-VL		40 Hz-WL vs. 40 Hz-VL		Baseline vs. Post-WL		Baseline vs. Post-VL		Post-WL vs. Post-VL	
	*t*_38_	*P*	*t*_38_	*p*	*t*_38_	*P*	*t*_38_	*p*	*t*_38_	*p*	*t*_38_	*p*
Delta	1.92	0.07	0.74	0.47	2.40	0.02 *	0.23	0.81	0.67	0.51	0.47	0.64
Theta	2.15	0.04 *	0.11	0.91	2.05	0.05 *	0.21	0.83	0.07	0.94	0.27	0.78
Alpha	0.91	0.37	0.28	0.78	0.62	0.53	0.52	0.60	0.34	0.73	0.19	0.86
Beta	1.56	0.14	0.69	0.51	0.83	0.41	0.96	0.35	1.08	0.29	0.19	0.86
Gamma	1.34	0.20	0.99	0.34	0.45	0.66	0.93	0.36	1.87	0.06	0.94	0.35

**Table 2:** Summary of statistical analysis results comparing averaged PSD for the prefrontal and occipital ROIs between 40 Hz-WL and 40 Hz-VL condition for each frequency band (* *p* < 0.05).

Frequency Band	Prefrontal Cortex 40 Hz-WL vs. 40 Hz-VL		Occipital Cortex 40 Hz-WL vs. 40 Hz-VL	
	*t*_38_	*p*	*t*_38_	*p*
Delta	1.89	0.07	1.87	0.04 *
Theta	2.03	0.04 *	1.34	0.18
Alpha	0.99	0.32	0.06	0.95
Beta	1.22	0.24	0.08	0.94
Gamma	1.18	0.24	0.30	0.77

**Table 3:** Summary of statistical analysis results comparing averaged PSD for the prefrontal and occipital ROIs between post-EEG with 40 Hz-WL and post-EEG with 40 Hz-VL for each frequency band (* *p* < 0.05).

Figure	Prefrontal Cortex		Occipital Cortex	
	*t*_38_	*p*	*t*_38_	*p*
Delta	0.34	0.73	0.45	0.66
Theta	0.25	0.81	0.23	0.81
Alpha	−0.03	0.98	0.15	0.87
Beta	−0.04	0.96	−0.22	0.82
Gamma	−0.53	0.60	−1.00	0.27

## Data Availability

Data sharing not applicable.

## Supplementary Materials

Page: 18. The following are available online at https://www.mdpi.com/article/10.3390/jpm11060557/s1, Figure S1: Schematic diagram of time of interest for PSD and VEP analyses. In the photo-stimulation experiment, 40 Hz light stimulation with a time width of 1 s was performed at 2-s intervals (i.e., 0.5 Hz) as shown in the figure above, and the EEG analysis was performed after epoching the continuous EEG data in the−1000 to 2000 ms segment. Specifically, the calculation of PSD was performed for the section indicated by the blue band, while the calculation of VEP was performed for the section indicated by the red band.
