# Supplementary material for: Photobiological Neuromodulation of Resting-State EEG and Steady-State Visual-Evoked Potentials by 40 Hz Violet Light Optical Stimulation in Healthy Individuals
Source: J Pers Med. 2021 Jun 15;11(6):557. doi: 10.3390/jpm11060557 (PMC8232632; doi:10.3390/jpm11060557)
Supplement: Supplementary file 1 [file jpm-11-00557-s001.zip › jpm-1198032-supplementary.pdf]

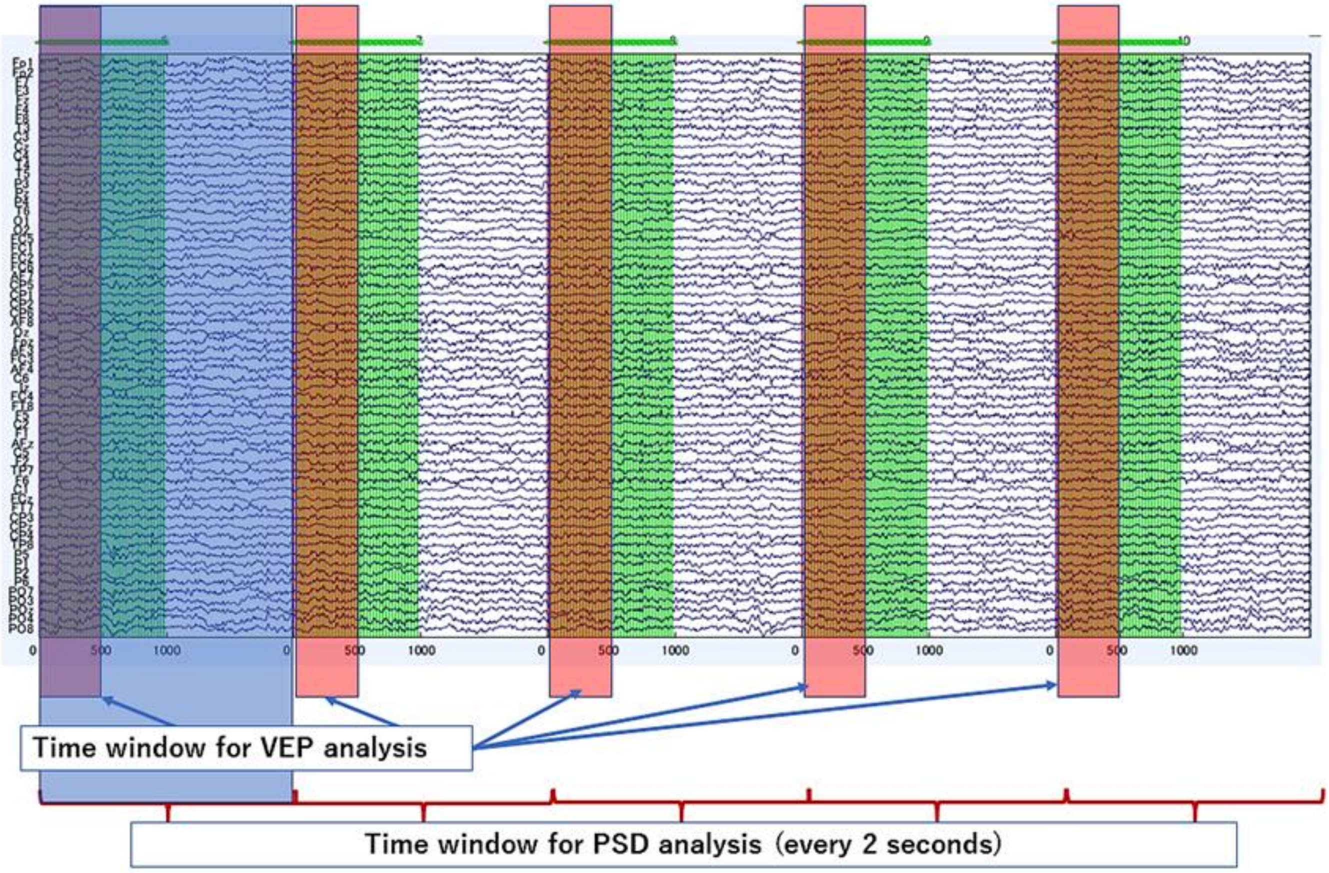

Figure S1. Schematic diagram of time of interest for PSD and VEP analyses. In the photo-stimulation experiment, 40 Hz light stimulation with a time width of 1 s was performed at 2-s intervals (i.e., 0.5 Hz) as shown in the figure above, and the EEG analysis was performed after epoching the continuous EEG data in the−1000 to 2000 ms segment. Specifically, the calculation of PSD was performed for the section indicated by the blue band, while the calculation of VEP was performed for the section indicated by the red band.
